# Associations of Genetic Variants in the *PSCA*, *MUC1* and *PLCE1* Genes with Stomach Cancer Susceptibility in a Chinese Population

**DOI:** 10.1371/journal.pone.0117576

**Published:** 2015-02-06

**Authors:** Hongwei Sun, Xiaoli Wu, Fang Wu, Ying Li, Zhengping Yu, Xiangrong Chen, Yunzhi Chen, Wenjun Yang

**Affiliations:** 1 Department of Surgery, First Affiliated Hospital, Wenzhou Medical University, Wenzhou 325000, Zhejiang, China; 2 Department of Gastroenterology, First Affiliated Hospital, Wenzhou Medical University, Wenzhou 325000, Zhejiang, China; 3 Department of Operating room, First Affiliated Hospital, Wenzhou Medical University, Wenzhou 325000, Zhejiang, China; Sanjay Gandhi Medical Institute, INDIA

## Abstract

**Background:**

Several genetic variants including *PSCA* rs2294008 C>T and rs2976392 G>A, *MUC1* rs4072037 T>C, and *PLCE1* rs2274223 A>G have shown significant association with stomach cancer risk in the previous genome-wide association studies (GWASs).

**Methods:**

To evaluate associations of these SNPs in the Han Chinese, an independent hospital based case-control study was performed by genotyping these four polymorphisms in a total of 692 stomach cancer cases and 774 healthy controls acquired by using frequency matching for age and gender. False-positive report probability (FPRP) analysis was also performed to validate all statistically significant findings.

**Results:**

In the current study, significant association with stomach cancer susceptibility was observed for all the four polymorphisms of interest. Specifically, a significant increased stomach cancer risk was associated with *PSCA* rs2294008 (CT vs. CC: adjusted OR = 1.37, 95% CI = 1.07–1.74, and CT/TT vs.CC: adjusted OR = 1.30, 95% CI = 1.03–1.63), *PSCA* rs2976392 (AG vs. GG: adjusted OR = 1.30, 95% CI = 1.02–1.65, and AG/AA vs. GG: adjusted OR = 1.26, 95% CI = 1.00–1.59), or *PLCE1* rs2274223 (AG vs. AA: adjusted OR = 1.48, 95% CI = 1.15–1.90, and AG/GG vs. AA: adjusted OR = 1.45, 95% CI = 1.14–1.84), respectively. In contrast, *MUC1* rs4072037 was shown to decrease the cancer risk (CT vs. TT: adjusted OR = 0.77, 95% CI = 0.60–0.98). Patients with more than one risk genotypes had significant increased risk to develop stomach cancer (adjusted OR = 1.30, 95% CI = 1.03–1.64), when compared with those having 0–1 risk genotypes. Stratified analysis indicated that the increased risk was more pronounced in younger subjects, men, ever smokers, smokers with pack years ≤ 27, patients with high BMI, or non-cardia stomach cancer.

**Conclusions:**

This study substantiated the associations between four previous reported genetic variants and stomach cancer susceptibility in an independent Han Chinese population. Further studies with larger sample size and different ethnicities are warranted to validate our findings.

## Introduction

Stomach cancer is the fourth most frequently diagnosed cancer and the second leading cause of cancer-related death worldwide, with approximately 738,000 cancer-related deaths in 2008. Generally, more than 70% of new stomach cancer cases and deaths occur in developing countries, with highest incidence rate in Eastern Asia. Particularly, approximately 40% of world’s stomach cancer cases have occurred in China [1,2]. *Helicobacter pylori* (*H*. *pylori*) infection is well-established etiologic factor for stomach cancer worldwide, with infection rates ranging from 40% to 80% in humans. Besides the *H*. *pylori* infection, salted and nitrated foods consumption, and cigarette smoking are also been reported to be associated with increased stomach cancer risk, whereas fresh fruits and vegetables intakes are recognized as protective factors [3]. High body mass index (BMI) has been also suggested as a risk factor for stomach cancer in western countries [4], but not in China [5]. Nevertheless, only a small fraction of individuals exposed to risk factors eventually develop stomach cancer in the lifetime [6], suggesting that genetic factors may play an important role in the pathogenesis of stomach cancer. To date, genetic etiology of stomach cancer, such as gene-gene, and gene-environment interactions, remains unclear.

Over the past years, genome-wide association studies (GWASs), high throughput genotyping technologies, have been a robust tool in the discovery of novel cancer susceptibility loci or genes across the whole genome [7]. Thus far, GWASs have successfully identified hundreds of genetic markers that are related to the susceptibility to diseases including stomach cancer [8]. We aimed to investigate single-nucleotide polymorphisms (SNPs) in *PSCA*, *MUC1*, and *PLCE1* genes in this study. *PSCA* gene (located on chromosome 8q24) encodes a prostate stem cell antigen (PSCA), a protein composed of 123 amino acid residues. PSCA belongs to the LY-6/Thy-1 family of cell surface antigens. It is highly expressed in normal prostate and further up-regulated in prostate cancer [9], as well as non-prostatic malignancies including gastric cancer [10]. PSCA plays a critical role in cell adhesion, proliferation, and survival [11]. *In vitro* experiments indicated that some *PSCA* variants (e.g., rs2294008T) might decrease the transcription of the host gene by modulating its upstream fragment [10]. A two-stage GWAS for stomach cancer conducted among Japanese and Korean populations demonstrated that *PSCA* rs2976392 G>A and rs2294008 C>T SNPs significantly increased stomach cancer risk [10]. The associations of *PSCA* SNPs with gastric cancer were also confirmed in Chinese populations [12–18]. Moreover, a two-stage GWAS among a Chinese population by Abnet et al. [19] recently identified two clusters of SNPs at 1q22 (*MUC1* rs4072037 T>C) and 10q23 (*PLCE1* rs2274223 A>G) and their associations with stomach cancer susceptibility [19]. Simultaneously, a three-stage GWAS in another Chinese population by Wang et al. [20] also observed the association with rs2274223 A>G SNP. Mucin 1 (MUC1) is a membrane-bound protein which can anchor to the apical surface of gastrointestinal epithelia through a transmembrane domain [21]. MUC1 plays an important role in mucosal lubrication, protection against pathogens, signal transduction, and cell-cell interaction [22,23]. The protective function of MUC1 against infection in normal epithelial cells was confirmed by both *in vitro* and *in vivo* experiments [24]. Additionally, *PLCE1* gene encodes phospholipase C. This protein product can catalyze the hydrolysis of polyphatidylinositol 4,5-bisphosphate (PIP2) into two critical second messengers: inositol 1,4,5-trisphosphate (Insl,4,5P3) and 4,5-diacylglycerol (DAG) [25], and thereby regulate cell motility, fertilization, and sensory transduction [26]. The associations of *MUC1* rs4072037 T>C and *PLCE1* rs2274223 A>G with stomach cancer risk have also been replicated in different ethnicities [27–31]. Nevertheless, the combined effects of all these four polymorphisms on stomach cancer risk have not been investigated.

In the current study, we genotyped these four GWAS-indentified SNPs and assessed their associations with stomach cancer in a hospital based case-control study, comprising 692 cases and 774 cancer-free controls.

## Methods

### Study population

This case-control study included 692 genetically unrelated ethnic Han Chinese patients and 774 cancer-free controls. All the cases were newly diagnosed and histopathologically confirmed primary stomach cancer patients, recruited from the Department of Gastroenterology, First Affiliated Hospital of Wenzhou Medical University between January 2010 and September 2013. Patients with interstitialoma, metastasized cancer from other organs and recurrent tumors were excluded. All controls were randomly selected from hospital visitors who accompanied patients to the hospital but not seeking for medical care at the same time period, genetically unrelated to the enrolled case subjects. They were frequency matched to the cases by age (± within 5 years) and sex. During the recruitment of research participants, each participant was scheduled for an interview with trained interviewers after a written informed consent was signed. Demographic data and environmental exposure history were collected, such as age, gender, ethnicity, smoking history, alcohol consumption and family history of cancer. Each participant donated approximately 5 mL blood, of which 2 mL was used for genomic DNA extraction. The response rate was approximately 95% for stomach cancer subjects and 92% for controls. This study was approved by the Clinical Research Ethics Committee of Wenzhou Medical University, and all of the participants provided a written informed consent for donating their biological samples. Never smokers referred to less than 100 cigarettes all life. Those who drank alcoholic beverages at least once a week for one year or more or regularly were defined as drinkers.

### Genotyping

Genomic DNA was extracted and treated as described previously [32]. We adopted Taqman real time PCR method to genotype the selected four SNPs in cases and controls using a 7900 HT sequence detector system (Applied Biosystems). Eight positive controls and eight negative controls were included in each 384 well plate to ensure the accuracy of genotyping results. We also repeatedly genotyped 10% of the samples, and the results were 100% concordant.

### Statistical analysis

The χ^2^ test was calculated to evaluate the differences in the distributions of allele and genotype frequencies, as well as demographic between the cases and controls. Goodness-of-fit χ^2^ test was used to test the Hardy–Weinberg equilibrium (HWE) in the controls. Crude and adjusted odds ratios (ORs) and their corresponding 95% confidence intervals (CIs) were calculated by univariate and multivariate logistic regression models, respectively. We further performed stratification analyses by age, gender, smoking/drinking status, pack-year, BMI, tumor site and TNM stage. We also performed false-positive report probability (FPRP) analysis for all statistically significant findings [33]. Briefly, we preset a FPRP threshold of 0.2 and a prior probability of 0.1 for a given association between selected SNP and stomach cancer risk. Statistical power was calculated for detecting an OR of 1.50/0.67 for alleles with a risk/protective effect. Association with FPRP value < 0.2 was declared as noteworthy [34]. All statistics were performed by using SAS software (version 9.1; SAS Institute, Cary, NC). All statistical tests were two-sided, and *P*<0.05 was considered as statistical significant.

## Results

### Study subjects

The demographic characteristics of the 692 stomach cancer cases and 774 controls were summarized in **Table 1**. There were no significant gender different between cases and controls (*P* = 0.944). However, the controls were more likely to be smokers (*P*<0.0001) and drinkers (*P* = 0.0006) when compared with the patients. The cases were more likely to have nutrient deficiencies and lower BMI (*P*<0.0001). Therefore, smoking status, pack-years, drinking status and BMI were adjusted for in the subsequent multivariate logistic regression analyses. Among all cases, 199 (28.76%) had cardia cancer and 493 (71.24%) had non-cardia cancer. Moreover, stomach cancers were staged according to the TNM staging system in the 7^th^ Edition of the AJCC [35]. As a result, 274 cases (39.60%) were designated as TNM stage I or II diseases, while 418 (60.40%) presented with TNM stage III or IV diseases.

**Table 1:** Frequency distribution of selected characteristics in stomach cancer cases and controls.

Variables	Cases (n = 692)		Controls (n = 774)		P^a^
	No	%	No.	%	
Age range, yr	24–85		23–87		0.855
Mean ± SD	59.2 ± 11.1		59.7 ± 11.3		
≤ 50	134	19.36	151	19.51	
51–60	225	32.51	242	31.27	
61–70	226	32.66	249	32.17	
>70	107	15.46	132	17.05	
Gender					0.944
Males	492	71.10	549	70.93	
Females	200	28.90	225	29.07	
Smoking status					<0.0001
Never	427	61.71	362	46.77	
Ever	265	38.29	412	53.23	
Pack-years					<0.0001
0	427	61.71	362	46.77	
≤ 27 (mean)	133	19.22	250	32.30	
> 27 (mean)	132	19.08	162	20.93	
Drinking status					0.0006
Yes	153	22.11	232	29.97	
No	539	77.89	542	70.03	
BMI					<0.0001
<18.5	53	7.66	5	0.65	
18.5–24.0	423	61.13	244	31.52	
>24.0	216	31.21	525	67.83	
Tumor site					
Cardia	199	28.76	/	/	
Non-cardia	493	71.24	/	/	
TNM stage					
I+II	274	39.60	/	/	
III+IV	418	60.40	/	/	

^a^ Two-sided *χ* ^*2*^ test for distributions between stomach cancer cases and controls.

### Association between selected SNPs and stomach cancer susceptibility

The genotype distributions of the four selected SNPs in all subjects were shown in **Table 2**. All the observed genotype distributions in controls were in agreement with HWE (*P* = 0.105 for rs2294008, *P* = 0.130 for rs2976392, *P* = 0.155 for rs2274223, and *P* = 0.735 for rs4072037).

**Table 2:** Logistic regression analysis of associations between the genotypes of *PSCA*, *MUC1*, *PLCE1* and stomach cancer susceptibility in a Chinese population.

Genotype	Cases (N = 692)	Controls (N = 774)	*P* ^a^	Crude OR (95% CI)	P	Adjusted OR (95% CI) ^b^	*P* ^b^
*PSCA* rs2294008							
CC	332 (46.53)	405 (52.33)	0.048^c^	1.00		1.00	
CT	309 (44.65)	297 (38.37)		1.31 (1.05–1.63)	0.015	1.37 (1.07–1.74)	0.012
TT	61 (8.82)	72 (9.30)		1.07 (0.74–1.54)	0.737	1.02 (0.67–1.55)	0.924
CT/TT	370 (53.47)	369 (47.67)	0.027^d^	1.26 (1.03–1.55)	0.027	1.30 (1.03–1.63)	0.027
*PSCA* rs2976392							
GG	319 (46.10)	403 (52.07)	0.058^c^	1.00		1.00	
AG	308 (44.51)	299 (38.63)		1.30 (1.05–1.62)	0.017	1.30 (1.02–1.65)	0.035
AA	65 (9.39)	72 (9.30)		1.14 (0.79–1.65)	0.482	1.10 (0.73–1.66)	0.649
AG/AA	373 (53.90)	371 (47.93)	0.023^d^	1.27 (1.03–1.56)	0.023	1.26 (1.00–1.59)	0.0499
*PLCE1* rs2274223							
AA	405 (58.53)	514 (66.41)	0.007^c^	1.00		1.00	
AG	254 (36.71)	226 (29.20)		1.43 (1.14–1.78)	0.002	1.48 (1.15–1.90)	0.002
GG	33 (4.77)	34 (4.39)		1.23 (0.75–2.02)	0.410	1.26 (0.73–2.19)	0.403
AG/GG	287 (41.47)	260 (33.59)	0.002^d^	1.40 (1.13–1.73)	0.002	1.45 (1.14–1.84)	0.002
*MUC1* rs4072037							
TT	528 (76.30)	553 (71.45)	0.055^c^	1.00		1.00	
CT	143 (20.66)	201 (25.97)		0.75 (0.58–0.95)	0.019	0.77 (0.60–0.98)	0.035
CC	21 (3.03)	20 (2.58)		1.10 (0.59–2.05)	0.765	1.09 (0.58–2.06)	0.780
CT/CC	164 (23.70)	221 (28.55)	0.035	0.78 (0.62–0.98)	0.035	0.80 (0.63–1.01)	0.060
Combined effect of risk genotypes							
0–1	288 (41.62)	369 (45.67)	0.020	1.00		1.00	
2–4	404 (58.38)	405 (52.33)		1.28 (1.04–1.57)	0.020	1.30 (1.03–1.64)	0.026

^a^ *χ* ^*2*^ test for genotype distributions between stomach cancer cases and controls.

^b^ Adjusted for age, sex, BMI, smoking and drinking status.

^c^ Additive models.

^d^ Dominant models.

As indicated in **Table 2**, all of these four selected polymorphisms were associated with stomach cancer susceptibility. When the *PSCA* rs2294008 CC genotype was used as the reference, the CT genotype and a combination of CT and TT genotypes were associated with an increased stomach cancer risk (adjusted OR = 1.37, 95% CI = 1.07–1.74 for CT, and adjusted OR = 1.30; 95% CI = 1.03–1.63 for CT/TT). A similar association with stomach cancer risk was also found for the *PSCA* rs2976392 G>A polymorphism (AG vs. GG: adjusted OR = 1.30, 95% CI = 1.02–1.65, and AG/AA vs. GG: adjusted OR = 1.26; 95% CI = 1.00–1.59). Moreover, the *PLCE1* rs2274223 A>G polymorphism was found to significantly increase stomach cancer risk under the homozygous model (AG vs. AA: adjusted OR = 1.48, 95% CI = 1.15–1.90), and dominant model (AG/GG vs. AA: adjusted OR = 1.45, 95% CI = 1.14–1.84). In contrast, *MUC1* rs4072037 T>C polymorphism was shown to significantly decreased stomach cancer susceptibility under the homozygous model (CT vs. TT: adjusted OR = 0.77, 95% CI = 0.60–0.98). Furthermore, we found that subjects with 2–4 risk genotypes (the risk genotype referred to CT/TT for rs2294008 C>T, AG/AA for rs2976392 G>A, AG/GG for rs2274223 A>G, and TT for rs4072037 T>C polymorphism) had significant increased risk (adjusted OR = 1.30, 95% CI = 1.03–1.64) when compared with those with only 0–1 risk genotypes.

### Stratification analysis

The association between variant genotypes and stomach cancer risk was further evaluated in stratification analysis by age, gender, smoking status, pack-year, drinking status, and BMI under a dominant genetic model (**Table 3**). We found that the *PSCA* rs2294008 CT/TT genotypes were associated with increased stomach cancer risk in younger subjects, light smokers, and subjects with non-cardia cancer, when compared to respective reference groups. With respect to the *PLCE1* rs2274223 A>G polymorphism, stratification analyses observed increased stomach cancer risk with the AG/GG genotypes in younger participants, women, never smokers, never drinkers, participants with high BMI, and subjects with cardia cancer or TNM stage III+IV diseases. While risk genotypes were combined, we found that the subjects with 2–4 risk genotypes were more likely to develop stomach cancer among younger subgroup, males, ever smokers, or subgroups with high BMI and subjects with non-cardia cancer, than each corresponding subgroup counterparts with 0–1 risk genotype. The further heterogeneity tests for stratified analysis did not detect any difference between subgroups by different co-variates, such as age, sex, and smoking status. Moreover, there was no statistical evidence of interaction between these selected SNPs and co-variates (age, sex, BMI, etc), either.

**Table 3:** Stratification analysis of *PSCA* rs2294008 C>T, *PLCE1* rs2274223 A>G and risk genotypes with stomach cancer susceptibility.

Variables	rs2294008 (cases/controls)		Adjusted OR ^a^ (95% CI)	*P* ^a^	rs2274223 (cases/controls)		Adjusted OR ^a^ (95% CI)	*P* ^a^	Risk genotypes (cases/controls)		Adjusted OR ^a^ (95% CI)	*P* ^a^
	CC	CT/TT			AA	AG/GG			0–1	2–4		
Median age, yr												
≤59	150/202	183/159	1.65 (1.18–2.28)	0.003	200/251	133/110	1.59 (1.13–2.25)	0.008	132/173	201/188	1.50 (1.08–2.09)	0.016
>59	172/203	187/210	1.02 (0.73–1.41)	0.931	205/263	154/150	1.31 (0.94–1.82)	0.114	156/196	203/217	1.12 (0.81–1.55)	0.493
Gender												
Females	90/111	110/114	1.33 (0.86–2.05)	0.204	122/165	78/60	1.96 (1.22–3.16)	0.006	78/97	122/128	1.32 (0.85–2.05)	0.222
Males	232/294	260/255	1.31 (1.00–1.72)	0.053	283/349	209/200	1.28 (0.97–1.68)	0.086	210/272	282/277	1.32 (1.00–1.73)	0.047
Smoking status												
Never	197/182	230/180	1.29 (0.94–1.76)	0.115	242/262	185/100	2.09 (1.50–2.92)	<0.0001	178/160	249/202	1.20 (0.88–1.65)	0.249
Ever	125/223	140/189	1.31 (0.93–1.85)	0.130	163/252	102/160	0.95 (0.67–1.35)	0.772	110/209	155/203	1.43 (1.01–2.02)	0.044
Pack-year												
0	197/182	230/180	1.29 (0.94–1.76)	0.115	242/262	185/100	2.09 (1.50–2.92)	<0.0001	178/160	249/202	1.20 (0.88–1.65)	0.249
≤ 27	54/137	79/113	1.97 (1.21–3.21)	0.007	84/154	49/96	0.91 (0.56–1.50)	0.719	47/127	86/123	2.02 (1.23–3.30)	0.005
> 27	71/86	61/76	0.89 (0.52–1.51)	0.655	79/98	53/64	0.84 (0.49–1.44)	0.522	63/82	69/80	1.04 (0.61–1.78)	0.875
Drinking status												
Never	254/279	285/263	1.25 (0.96–1.64)	0.100	314/370	225/172	1.47 (1.12–1.95)	0.006	227/254	312/288	1.23 (0.94–1.61)	0.133
Ever	68/126	85/106	1.49 (0.95–2.35)	0.086	91/144	62/88	1.37 (0.86–2.18)	0.188	61/115	92/117	1.58 (1.00–2.50)	0.051
BMI												
<18.5	27/2	26/3	0.66 (0.09–4.98)	0.689	30/4	23/1	3.12 (0.30–32.55)	0.341	25/2	28/3	0.80 (0.11–5.79)	0.821
18.5–24.0	199/135	224/109	1.38 (1.00–1.90)	0.048	253/162	170/82	1.30 (0.93–1.82)	0.119	178/121	245/123	1.33 (0.97–1.83)	0.079
>24.0	96/268	120/257	1.35 (0.97–1.86)	0.074	122/348	94/177	1.57 (1.13–2.19)	0.008	85/246	131/279	1.41 (1.01–1.95)	0.043
Tumor site												
Cardia	106/405	93/369	1.01 (0.72–1.42)	0.963	92/514	107/260	2.18 (1.54–3.08)	<0.0001	92/369	107/405	1.12 (0.80–1.58)	0.507
Non-cardia	216/405	277/369	1.41 (1.10–1.82)	0.008	313/514	180/260	1.25 (0.96–1.63)	0.104	196/369	297/405	1.36 (1.05–1.75)	0.020
TNM stage												
I+II	123/405	151/369	1.36 (1.00–1.84)	0.047	172/514	102/260	1.29 (0.94–1.76)	0.120	110/369	164/405	1.35 (0.99–1.83)	0.057
III+IV	199/405	219/369	1.23 (0.95–1.61)	0.122	233/514	185/260	1.59 (1.21–2.09)	0.0009	178/369	240/405	1.24 (0.95–1.62)	0.112

^a^ Adjusted for age, sex, BMI, smoking and drinking status.

The FPRP values for all statistically significant result are shown in **Table 4**. False-positive report probability values for associations between stomach cancer risk and the frequency of genotypes of selected genes. **4**, with a preset prior probability of 0.1 and a FPRP threshold of 0.2. FPRP analysis indicated that the significant association between *PSCA* rs2294008 C>T and stomach cancer risk was noteworthy under homozygous model. Moreover, the association was also deserving of attention for younger subjects and those with non-cardia. Likewise, the significant association with *PLCE1* rs2274223 G>A was noteworthy for all subjects, as well as for younger subjects, never smokers, never drinkers, those with BMI >24.0, cardia cancer or TNM stage III+IV diseases. FPRP also confirmed the significant association with *PSCA* rs2976392 G>A under homozygous and dominant models and the significant association with *MUC1* rs4072037 T>C under homozygous model. As to the combined genotypes, we confirmed the significant association for the subjects with pack-year ≤27 or non-cardia cancer. Relatively greater FPRP values were found for the rest of significant associations between selected polymorphisms and stomach cancer risk, which might be ascribed to the relative small sample size of this study as well as moderate effects of selected SNPs. These findings need further validation in investigations with large sample size.

**Table 4:** False-positive report probability values for associations between stomach cancer risk and the frequency of genotypes of selected genes.

Genotype	Crude OR (95% CI)	*P* ^a^	Statistical power^b^	Prior probability				
				0.25	0.1	0.01	0.001	0.0001
*PSCA* rs2294008								
CT vs. CC	1.31 (1.05–1.63)	0.015	0.919	0.046	0.127	0.615	0.941	0.994
CT/TT vs. CC	1.26 (1.03–1.55)	0.027	0.951	0.078	0.202	0.736	0.966	0.996
CT/TT vs. CC								
≤59	1.65 (1.18–2.28)	0.003	0.353	0.025	0.071	0.457	0.895	0.988
Males	1.29 (1.01–1.65)	0.039	0.885	0.118	0.286	0.815	0.978	0.998
Pack-year ≤27	1.77 (1.16–2.72)	0.008	0.225	0.101	0.252	0.787	0.974	0.997
Non-cardia	1.41 (1.12–1.77)	0.003	0.710	0.013	0.039	0.308	0.818	0.978
Stage I+II	1.35 (1.02–1.78)	0.035	0.777	0.118	0.287	0.816	0.978	0.998
*PSCA* rs2976392								
AG vs. GG	1.30 (1.05–1.62)	0.017	0.927	0.052	0.142	0.646	0.949	0.995
AG/AA vs. GG	1.27 (1.03–1.56)	0.023	0.943	0.067	0.177	0.703	0.960	0.996
*PLCE1* rs2274223								
AG vs. AA	1.43 (1.14–1.78)	0.002	0.697	0.007	0.021	0.194	0.709	0.961
AG/GG vs. AA	1.40 (1.13–1.73)	0.002	0.739	0.007	0.021	0.194	0.709	0.961
AG/GG vs. AA								
≤59	1.52 (1.11–2.08)	0.009	0.472	0.055	0.148	0.656	0.951	0.995
Females	1.76 (1.17–2.65)	0.007	0.220	0.087	0.223	0.759	0.970	0.997
Males	1.29 (1.00–1.65)	0.046	0.888	0.135	0.319	0.837	0.981	0.998
Never smoker	2.00 (1.48–2.70)	<0.0001	0.027	0.001	0.002	0.021	0.179	0.686
Never drinker	1.54 (1.20–1.98)	0.001	0.422	0.005	0.015	0.141	0.624	0.943
BMI >24	1.52 (1.10–2.10)	0.012	0.478	0.070	0.184	0.713	0.962	0.996
Cardia	2.30 (1.68–3.15)	<0.0001	0.004	0.000	0.000	0.005	0.046	0.325
III+IV	1.57 (1.23–2.00)	0.0003	0.362	0.002	0.007	0.076	0.453	0.892
*MUC1* rs4072037								
CT vs. TT	0.75 (0.58–0.95)	0.019	0.795	0.066	0.175	0.701	0.959	0.996
CT/CC vs. TT	0.78 (0.62–0.98)	0.035	0.881	0.106	0.263	0.797	0.975	0.997
Combined risk genotypes								
2–4 vs. 0–1	1.28 (1.04–1.57)	0.020	0.933	0.060	0.161	0.679	0.955	0.995
2–4 vs. 0–1								
≤59	1.40 (1.04–1.89)	0.028	0.670	0.112	0.275	0.807	0.977	0.998
Male	1.32 (1.03–1.68)	0.027	0.847	0.087	0.222	0.758	0.969	0.997
Ever smoker	1.45 (1.06–1.98)	0.019	0.584	0.090	0.228	0.765	0.970	0.997
Pack-year ≤27	1.89 (1.23–2.91)	0.004	0.155	0.072	0.189	0.719	0.963	0.996
Non-cardia	1.38 (1.10–1.74)	0.006	0.760	0.022	0.064	0.430	0.884	0.987
Stage I+II	1.36 (1.03–1.80)	0.032	0.756	0.112	0.275	0.807	0.977	0.998

^a^ Chi-square test was performed to calculate the genotype frequency distributions.

^b^ Statistical power was calculated using the number of observations in the subgroup and the OR and *P* values in this table.

## Discussion

In the current hospital based case-control study, we investigated the potential associations of *PSCA* rs2294008 C>T and rs2976392 G>A, *PLCE1* rs2274223 A>G and *MUC1* rs4072037 T>C polymorphisms with stomach cancer susceptibility among a Chinese population. We found that *PSCA* rs2294008 CT/TT, *PSCA* rs2976392 AG/AA and *PLCE1* rs2274223 AG/GG genotypes were associated with a significantly increased stomach cancer risk in a Chinese population, whereas, the *MUC1* rs4072037 T>C were associated with decreased stomach cancer susceptibility. We also found that subjects carrying 2–4 risk genotypes had a pronouncedly increased stomach cancer risk, when compared to those carrying 0–1 risk genotype. The effect of combined risk genotypes on cancer risk was more evident in younger subjects, males, ever smokers, subjects with high BMI and non-cardia stomach cancer. These findings indicate that the selected SNPs from GWASs may contribute to stomach carcinogenesis.

So far, four GWASs have investigated stomach cancer risk [10,16,19,20] as mentioned in the Introduction, which led to the finding of the four SNPs of interest. The *PSCA* rs2294008 C>T results in Met/Thr substitution, and the *PSCA* rs2976392 G>A may alter transcription factor binding site activity of the gene [10]. Moreover, *MUC1* rs4072037 T>C may lead to splicing alteration, whereas, *PLCE1* rs2274223 A>G may cause an Arg-to-His change that were significantly associated with risk of stomach cancer in the initial scanning phase [19]. While scanning and validation phases were combined, a genome-wide association was observed only for the *PLCE1* rs2274223 A>G polymorphism, but not the *MUC1* rs4072037 T>C polymorphism [19]. Simultaneously, Wang et al. also found the rs2274223 polymorphism was associated with gastric cardia adenocarcinoma (*P* = 1.74×10^–39^) [20]. Most recently, GWAS by Shi et al. [16], confirmed previously reported associations of non-cardia gastric cancer susceptibility with not only *PSCA* rs2294008 and rs2976392, but also *MUC1* rs4072037.

The findings from previous GWASs were widely validated among different ethnic populations in recent years (**S1 Table**). For example, Wu et al. [18] indicated that the association between *PSCA* rs2294008 and stomach cancer was more prominent among patients with non-cardia stomach cancer than those with cardia stomach cancer. The significant association was also validated by studies conducted among different ethnicities worldwide [14–17,19,36–40]. However, the association between rs2294008 C>T and stomach cancer was not validated by others [12,41]. To resolve the controversy, six meta-analyses have been performed to evaluate the relationship between *PSCA* polymorphisms and gastric cancer susceptibility [42–47]. Qiao et al. [42] included eight case-control studies from seven articles and found that rs2294008 T allele and rs2976392 A allele were significantly associated with increased gastric cancer risk. These findings were also confirmed by other meta-analysis [43–46]. More recently, to access the contributions of these two widely investigated *PSCA* SNPs to gastric cancer susceptibility, Gu et al. [47] performed a meta-analysis of 16 studies with a total of 18,820 cases and 35,756 controls. The pooled OR was 1.46 (95% CI = 1.30–1.69) for the *PSCA* rs2294008 and 1.49 (95% CI = 1.22–1.82) for rs2976392 polymorphisms. Moreover, after discovered by Abnet et al. [19] and Wang et al. [23], the *PLCE1* rs2274223 polymorphism have been extensively investigated among different ethnicities in different cancers, such as stomach cancer, esophageal cancer, head and neck cancer, and gallbladder cancer [48–60]. However, the conclusions on the association between the *PLCE1* rs2274223 A>G polymorphism and cancer risk are controversial. The significant association was observed in some studies [49–52,56,58], but not in others [48,53–55,57,59,60]. Four meta-analyses were performed to re-evaluate the association [27–30]. Hao et al. [27] included a total of 13 case-control studies, of which five studies with 5127 cases and 5791 controls examined the role of this SNP in gastric cancer risk. They found statistically significant associations between the rs2274223 polymorphism and increased gastric cancer risk under the homozygous model and heterozygous model. These results were consistent with those of other three meta-analyses that included fewer association studies on gastric cancer. As to the *MUC1* rs4072037 T>C polymorphism, the association between this polymorphism and gastric cancer was validated among different ethnicities [49,53,61]. Saeki et al. [61] and Zhang et al. [49] found that this polymorphism was associated with decreased stomach cancer among Asians, while no significant association was found among Caucasians [53].There was only one meta-analysis for *MUC1* rs4072037 T>C polymorphism [31], in which a total of 10 studies with 6580 gastric cancer cases and 10324 controls were included. It was found that the *MUC1* rs4072037 G allele was significantly associated with a decreased gastric cancer risk (OR = 0.72, 95% CI = 0.68–0.77), when compared with the A allele.

Numerous studies have been carried out to validate the GWAS findings on stomach cancer. Nevertheless, none of studies covered all of the four SNPs as we did here, except for one study conducted by Palmer et al. among Caucasians, which investigated *PLCE1* rs2274223, *C20orf54* rs13042395 and *MUC1* rs4072037 polymorphisms [53]. They found that the *MUC1* rs4072037 polymorphism was associated with a decreased risk of intestinal-type gastric cancer (OR = 0.4, 95% CI = 0.2–0.9); however, no associations were found with both the *PLCE1* rs2274223 and *C20orf54* rs13042395. In the current study, we found all of these four SNPs were individually associated with stomach cancer susceptibility among Chinese subjects. We also found that 2–4 risk genotype carriers had a much higher stomach cancer risk than the 0–1 carriers. This phenomenon was more pronounced in younger subjects, males, ever smoker, those with high BMI, and subjects with non-cardia stomach cancer. Cigarette smoke contains about 55 carcinogens that can generate reactive oxygen species to induce a variety of DNA damages. Male ever smokers consistently exposed to cigarettes smoke may possibly harbor DNA damages that can interact with genetic variations to lead to cancer development. In other words, gene-environment interaction may play important roles in initiating and promoting carcinogenesis [62]. High BMI has been recognized as a risk factor for stomach cancer in western countries [4]. Cardia stomach cancer is localized to the gastroesophageal junction and may differ from non-cardia cancer regarding epidemiological characteristics and clinical features [16].Therefore, the association with non-cardia stomach cancer appeared to be biology plausible.

In summary, we confirmed the associations between four previous GWAS-indentified SNPs and stomach cancer susceptibility in this hospital based case-control study. However, several limitations in the present study should be addressed. First, the inherent selection bias and information bias may be inevitable in this hospital based case-control designed study. Second, we only included four SNPs in the current study, instead of covering all promising GWAS-indentified SNPs. Generally, studies comprising more SNPs potentially related to stomach cancer risk may be more capable of illuminating the exact role of genetic variants in stomach carcinogenesis. Finally, due to the nature of retrospective study design, we did not have reliable and sufficient information for individuals on other environmental exposures, such as *H*. *pylori* infection, dietary, occupation exposure, as well as stomach cancer classification and subtypes, such as intestinal and diffuse subtype. Lack of all the valuable information hindered us to further investigate the etiological roles of these factors in the stomach carcinogenesis. Despite these limitations, the findings from our study were informative for researchers and physicians in this field. Additional well-designed prospective population-based studies are needed to further confirm our findings, particularly those with detailed information on the risk factors for stomach cancer and large sample size including different ethnic groups.

## Supporting Information

S1 Data Original Data. (XLS)

S1 Table Characteristics of previous studies focused on these four SNPs. (DOC)
