# Supplementary material for: Associations of Genetic Variants in the PSCA, MUC1 and PLCE1 Genes with Stomach Cancer Susceptibility in a Chinese Population
Source: PLoS One. 2015 Feb 6;10(2):e0117576. doi: 10.1371/journal.pone.0117576 (PMC4319726; doi:10.1371/journal.pone.0117576)
Supplement: S1 Table — (DOC) [file pone.0117576.s002.doc]

| **Supplemental Table 1.** Characteristics of previous studies focused on these four SNPs | | | | | | | | | |
| --- | --- | --- | --- | --- | --- | --- | --- | --- | --- |
| Surname | Year | Cancer | Country | Ethnicity | Source | Genotype method | Case | Control | Main results |
| ***PSCA* rs2294008 C>T polymorphism** | | | | | | | | | |
| Sakamoto | 2008 | gastric | Japan | Asian | HB | GWAS | 1524 | 1396 | association |
| Sakamoto | 2008 | gastric | Korea | Asian | HB | Taqman | 871 | 390 | association |
| Matsuo | 2009 | gastric | Japan | Asian | HB | Taqman | 708 | 708 | association |
| Wu | 2009 | gastric | China | Asian | PB | PCR-RFLP | 1710 | 995 | association |
| Wu | 2009 | bladder | USA&European | Caucasian | HB | GWAS | 5038 | 9363 | association |
| Abnet | 2010 | gastric | China | Asian | PB | GWAS | 2100 | 1625 | association |
| Lu | 2010 | gastric | China | Asian | PB | PCR-RFLP | 1023 | 1069 | no association |
| Ou | 2010 | gastric | China | Asian | HB | PCR/LDR | 196 | 246 | association |
| Wang | 2010 | bladder | China | Asian | HB | PCR-RFLP | 581 | 580 | association |
| Joung | 2011 | prostate | Korea | Asian | HB | MassARRAY | 192 | 168 | no association |
| Lochhead | 2011 | gastric | Poland | Caucasian | PB | Taqman | 292 | 382 | association |
| Lochhead | 2011 | gastric | USA | Caucasian | PB | Taqman | 308 | 208 | association |
| Lochhead | 2011 | EAC | USA | Caucasian | PB | Taqman | 107 | 208 | association |
| Lochhead | 2011 | ESCC | USA | Caucasian | PB | Taqman | 51 | 208 | association |
| Shi | 2011 | gastric | China | Asian | HB | GWAS | 4294 | 5882 | association |
| Song | 2011 | gastric | Korea | Asian | HB | PCR-RFLP | 3245 | 1700 | association |
| Zeng | 2011 | gastric | China | Asian | HB | PCR-RFLP | 460 | 549 | association |
| Fu | 2012 | bladder | European&USA | Caucasian | PB | GWAS | 5393 | 7324 | association |
| Kim | 2012 | breast | Korea | Asian | HB | MassARRAY | 451 | 459 | no association |
| Li | 2012 | gastric | China | Asian | PB | MassARRAY | 300 | 300 | association |
| Sala | 2012 | gastric | European | Caucasian | PB | Taqman | 409 | 1515 | association |
| Smith | 2012 | colorectal | UK | Caucasian | HB | Taqman | 77 | 804 | no association |
| Ma | 2013 | bladder | China | Asian | PB | MassARRAY | 175 | 962 | no association |
| Ono | 2013 | gallbladder | Japan | Asian | HB | Taqman | 44 | 173 | no association |
| Rai | 2013 | gallbladder | India | Asian | HB | Taqman | 405 | 247 | no association |
| Rizzato | 2013 | gastric | Germany | Caucasian | PB | Taqman | 178 | 1057 | association |
| Zhao | 2013 | gastric | China | Asian | PB | DHPLC | 717 | 951 | association |
| Dai | 2014 | ESCC | China | Asian | PB | Taqman | 2083 | 2220 | association |
| Sun | 2014 | gastric | USA | African | HB | Taqman | 130 | 125 | association |
| Wang | 2014 | bladder | China | Asian | PB | Taqman | 1210 | 1008 | association |
| Current (Sun) |  | gastric | China | Asian | HB | Taqman | 692 | 774 | association |
| ***PSCA* rs2976392 G>A polymorphism** | | | | | | | | | |
| Sakamoto | 2008 | gastric | Japan | Asian | HB | GWAS | 1525 | 1397 | association |
| Sakamoto | 2008 | gastric | Korea | Asian | HB | Taqman | 865 | 390 | association |
| Matsuo | 2009 | gastric | Japan | Asian | HB | Taqman | 707 | 707 | association |
| Wu | 2009 | gastric | China | Asian | PB | PCR-RFLP | 1724 | 1002 | association |
| Lu | 2010 | gastric | China | Asian | PB | PCR-RFLP | 1043 | 1082 | association |
| Ou | 2010 | gastric | China | Asian | HB | PCR/LDR | 196 | 246 | no association |
| Joung | 2011 | prostate | Korea | Asian | HB | MassARRAY | 194 | 168 | no association |
| Shen | 2011 | gastric | China | Asian | PB | DHPLC | 60 | 60 | association |
| Shi | 2011 | gastric | China | Asian | HB | GWAS | 4294 | 5882 | association |
| Kim | 2012 | breast | Korea | Asian | HB | MassARRAY | 453 | 460 | no association |
| Ono | 2013 | gallbladder | Japan | Asian | HB | Taqman | 44 | 173 | no association |
| Ju | 2013 | gastric | China | Asian | HB | sequencing | 155 | 210 | association |
| Wang | 2014 | gastric | China | Asian | HB | Taqman | 283 | 275 | no association |
| Current (Sun) |  | gastric | China | Asian | HB | Taqman | 692 | 774 | association |
| ***PLCE1* rs2274223 A>G polymorphism** | | | | | | | | | |
| Abnet | 2010 | Gastric | China | Asian | PB | Illumina | 1625 | 2100 | association |
| Abnet | 2010 | ESCC | China | Asian | PB | Illumina | 1898 | 2100 | association |
| Wang | 2010 | Gastric | China | Asian | HB | Illumina | 2766 | 11013 | association |
| Wang | 2010 | ESCC | China | Asian | HB | Illumina | 8750 | 12746 | association |
| Ma | 2011 | SCCHN | USA | Caucasian | HB | Taqman | 1097 | 1089 | no association |
| Shi | 2011 | gastric | China | Asian | HB | GWAS | 4294 | 5882 | no association |
| Zhang | 2011 | gastric | China | Asian | PB | Taqman | 1665 | 1848 | association |
| Bye | 2012 | ESCC | South African | African | Mixed | Taqman | 418 | 850 | no association |
| Bye | 2012 | ESCC | South African | Mixed | HB | Taqman | 254 | 857 | no association |
| Gu | 2012 | ESCC | China | Asian | HB | MassArray | 379 | 371 | association |
| Hu | 2012 | ESCC | China | Asian | HB | Taqman | 1061 | 1211 | association |
| Li | 2012 | colorectal | China | Asian | HB | MassArray | 231 | 292 | association |
| Yang | 2012 | gastric | China | Asian | HB | MassArray | 249 | 292 | no association |
| Palmer | 2012 | gastric | Poland | Caucasian | PB | Taqman | 289 | 376 | no association |
| Palmer | 2012 | gastric | USA | Caucasian | PB | Taqman | 306 | 210 | no association |
| Palmer | 2012 | ESCC | USA | Caucasian | PB | Taqman | 52 | 210 | association |
| Palmer | 2012 | EAC | USA | Caucasian | PB | Taqman | 107 | 210 | No association |
| Wang | 2012 | gastric | China | Asian | HB | Taqman | 1059 | 1240 | association |
| Zhou | 2012 | ESCC | China | Asian | HB | PCR-LDR | 517 | 510 | association |
| Dura | 2013 | ESCC | Netherlands | Caucasian | PB | Taqman | 86 | 580 | no association |
| Dura | 2013 | EAC | Netherlands | Caucasian | PB | Taqman | 258 | 580 | no association |
| Duan | 2013 | ESCC | China | Asian | PB | PCR-RFLP | 381 | 420 | association |
| Li | 2013 | gastric | China | Asian | HB | Taqman | 335 | 334 | no association |
| Yuan | 2013 | head&neck | China | Asian | HB | Taqman | 501 | 879 | association |
| Sharma | 2013 | gallbladder | Indian | Caucasian | HB | PCR-RFLP | 416 | 225 | association |
| Chen | 2013 | ESCC | China | Asian | HB | MALDI-TOF MS | 200 | 300 | association |
| Malic | 2014 | esophageal | India | Asian | HB | PCR-RFLP | 135 | 195 | no association |
| Wang | 2014 | colorectal | China | Asian | HB | Taqman | 417 | 416 | association |
| Song | 2014 | gastric | Korea | Asian | HB | Taqman | 3245 | 1700 | no association |
| Umar | 2014 | ESCC | India | Asian | HB | PCR-RFLP | 293 | 314 | no association |
| Current (Sun) |  | gastric | China | Asian | HB | Taqman | 692 | 774 | association |
| ***MUC1* rs4072037 T>C polymorphism** | | | | | | | | | |
| Strawbridge | 2008 | prostate | Sweden | Caucasian | PB | Beckman | 130 | 67 | association |
| Kruit | 2008 | breast | Netherlands | Caucasian | HB | Taqman | 162 | 208 | no association |
| Xu | 2009 | gastric | China | Asian | PB | PCR-SSPs | 138 | 241 | association |
| Jia | 2010 | gastric | Poland | Caucasian | PB | SNPlex | 272 | 376 | association |
| Abnet | 2010 | gastric | China | Asian | PB | Illumina | 2240 | 3302 | association |
| Saeki | 2011 | gastric | Korea | Asian | PB | PCR-LDR | 449 | 369 | association |
| Saeki | 2011 | gastric | Japan | Asian | PB | Taqman | 303 | 1465 | association |
| Saeki | 2011 | gastric | Japan | Asian | PB | Taqman | 605 | 1264 | association |
| Shi | 2011 | gastric | China | Asian | HB | GWAS | 4294 | 5882 | association |
| Zhang | 2011 | gastric | China | Asian | PB | Taqman | 1658 | 1833 | association |
| Palmer | 2012 | gastric | USA | Caucasian | PB | Taqman | 311 | 207 | association |
| Palmer | 2012 | ESCC | USA | Caucasian | PB | Taqman | 52 | 207 | association |
| Palmer | 2012 | EAC | USA | Caucasian | PB | Taqman | 107 | 207 | no association |
| Li | 2012 | colorectal | China | Asian | HB | MassArray | 231 | 292 | no association |
| Yang | 2012 | gastric | China | Asian | HB | MassArray | 249 | 292 | association |
| Li | 2013 | gastric | China | Asian | HB | Taqman | 335 | 334 | association |
| Song | 2014 | gastric | Korea | Asian | HB | Taqman | 3225 | 1697 | association |
| Williams | 2014 | ovarian | USA | Caucasian | PB | Taqman | 727 | 757 | no association |
| Current (Sun) |  | gastric | China | Asian | HB | Taqman | 692 | 774 | association |
| SCCHN, squamous cell carcinoma of head and neck; ESCC, esophageal squamous cell carcinoma; EAC, esophageal adenocarcinoma; HB, hospital based; PB, population based; GWAS, genome wide association study; PCR-RFLP, polymorphism chain reaction-restriction fragment length polymorphism; PCR-LDR, polymorphism chain reaction-ligase detection reaction; DHPLC, denaturing high performance liquid chromatography; PCR-SSPs, sequence-specific primers-polymerase chain reaction. | | | | | | | | | |
